# Wearable Sensing Systems for Monitoring Mental Health

**DOI:** 10.3390/s22030994

**Published:** 2022-01-27

**Authors:** Mijeong Kang, Kyunghwan Chai

**Affiliations:** Department of Cogno-Mechatronics Engineering, College of Nanoscience & Nanotechnology, Pusan National University, Busan 46241, Korea; 201810333@pusan.ac.kr

**Keywords:** mental healthcare, mental stresses, wearable sensors, flexible sensors

## Abstract

Wearable systems for monitoring biological signals have opened the door to personalized healthcare and have advanced a great deal over the past decade with the development of flexible electronics, efficient energy storage, wireless data transmission, and information processing technologies. As there are cumulative understanding of mechanisms underlying the mental processes and increasing desire for lifetime mental wellbeing, various wearable sensors have been devised to monitor the mental status from physiological activities, physical movements, and biochemical profiles in body fluids. This review summarizes the recent progress in wearable healthcare monitoring systems that can be utilized in mental healthcare, especially focusing on the biochemical sensors (i.e., biomarkers associated with mental status, sensing modalities, and device materials) and discussing their promises and challenges.

## 1. Introduction

Globally, nearly two billion people suffer from mental health problems, which are attributed to brain function impairment and characterized by abnormal perceptions, thoughts, emotions, behavior, and/or social interactions. It is predicted that the global cost for managing mental problems will rise to 16 trillion USD by 2030 [1]. The importance of mental healthcare, however, has been relatively underestimated compared to that of the treatments of physical and physiological illnesses [2,3]. Mental health services are under-resourced in many countries and have worsened since the COVID-19 outbreak that inevitably exhausts clinical personnel and resources. We are witnessing the consequential impacts on COVID-19 patients, frontline workers, those with existing mental illnesses, and general populations [4,5,6]. The increased needs of mental healthcare have brought more attention to the developments of sensors for monitoring mental status, which are easily accessible to everyone, every time, and everywhere.

**Figure 1:**
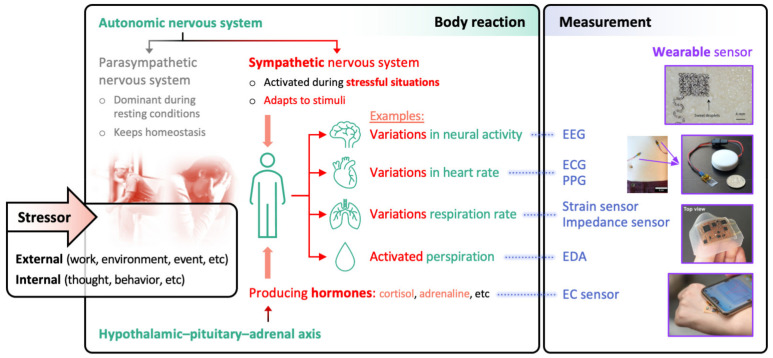
Physiological reactions to stressors and representative methods for measuring the stress reactions. EEG, electroencephalography; ECG, electrocardiograph; PPG, photoplethysmography; EDA, electrodermal activity; and EC, electrochemical.

Stress is the response of a human body to stressors, that is, any physical, cognitive, or emotional challenges, to cope with demanding situations (Figure 1). This response is orchestrated by autonomic nervous system (ANS) and hypothalamic–pituitary–adrenal (HPA) axis, which are interconnected [7]. The ANS has two branches: sympathetic nervous system (SNS) that quickly excites some vital organs (e.g., heart, lungs, sweat glands) and parasympathetic nervous system (PNS) that inhibits such excitation. They complementarily work to modulate vital body functions, maintaining physiological homeostasis. Under stressful condition, the SNS is activated and consequently heart rate increases, respiration rate increases, sweat is secreted, and other physiological systems are affected, which helps the individual to fight against or fly from the challenges. In addition, the HPA axis, which is a hormonal system, is initiated in response to the stressor and drives a series of endocrine events, ending up with the secretion of cortisol. Cortisol affects several physiological systems to support the fight-or-flight response (e.g., by suppressing immune responses and activating metabolisms), and also enhances the SNS-mediated physiological changes. Observing the activities of SNS and HPA axis, therefore, provides the opportunity to access the health status, including mental state.

Short-lasting daily stressors trigger acute stress, and our body is usually resilient to such stress; excessive and/or prolonged stress, however, damages the body, resulting in physiological illnesses (e.g., high blood pressure) and can increase the risk of developing mental problems (e.g., depression) [8]. To prevent the severe mental problems by timely carrying out appropriate stress management, it is critical to objectively monitor the physiological and biochemical signs associated with the SNS and HPA axis and ultimately associated with stress, in a continuous or on-demand manner. Currently, stress level measurement largely relies on subjective, time-consuming methods, mainly clinical interviews and self-reporting questionnaires. These methods require trained clinicians for data collection and interpretation, inevitably limiting their accessibility. And these methods are subject to reporting unreliable results due to personal bias, possibly confounding the subsequent clinical decision making for mental health treatments [9,10]. In this context, there has been an increasing interest in wearable sensors that can collect in real time the biosignals associated with mental status [11]. This review gives an overview of recent developments in several types of wearable sensors, with more focus on sensor materials, that can detect cardiac, respiratory, and perspiratory activities and/or molecular secretion, which can be utilized to support mental health management.

## 2. Wearable Sensors for Monitoring Cardiac Activity

**Figure 2:**
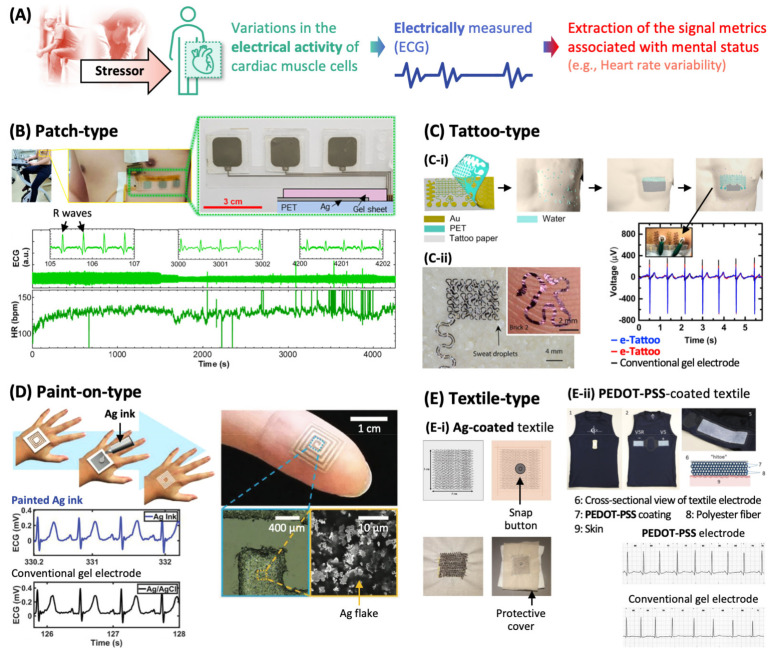
Wearable sensors for monitoring cardiac activities. (**A**) Mental stress-relevant cardiac activities can be monitored by ECG. (**B**) Gel electrode patch, (**C**) e-tattoo, (**D**) sprayed electronics, and (**E**) conductive textiles for the measurement of electrical potential generated by cardiac muscle cells. ECG, electrocardiography; PET, poly(ethylene terephthalate); and PEDOT-PSS, poly(ethylenedioxythiophene):poly(styrenesulfonate).

Stressors induce changes in the myocardial activity facilitating blood distribution to vital organs, and this can be quantified with heart rate (i.e., the number of heartbeats per minute), stroke volume (i.e., the blood volume pumped by a single heartbeat), and the vascular activity (i.e., the contraction and dilation of blood vessels changing the local blood pressure). Heartbeat is one of the most generally used biosignals for examining the physical and mental status. Among the metrics that characterize heartbeat, heart rate variability (HRV, beat-to-beat variability) is used as a clinical sign of mental stress due to its high correlation with the ANS and several mental disorders (Figure 2A) [12]. For instance, lowered HRV is indicative of impaired ANS homeostasis and therefore reduced ability to cope with stressors, and reported to be associated with anxiety, depression, and panic disorder [13].

There are several approaches for monitoring myocardial activity. One robust technology is electrocardiography (ECG), which measures the activity of heart by recording the electrical potential generated by cardiac muscle cells using the electrodes attached to the body. To “accurately” monitor ECG signals in the “long term”, the performances of ECG sensors have been improved along with the development of functional materials and device designs [14]. From the standpoint of signal quality, it is essential to reduce the electrical impedance in the electrode–skin interface by securing the tight contact between them, and it is required to eliminate the electrical noises mainly generated from electric wires connecting the electrodes and the measuring instrument. In terms of user-friendliness (e.g., to monitor ECG signals continuously during daily routines for more than few weeks), the electrode–skin contact need to be comfortable, which requires appropriate chemical and mechanical properties of the electrode material.

Wearable ECG sensors with advanced materials have a great potential for the continuous monitoring of cardiac activities, and there are various types of ECG sensors. Figure 2B shows a typical patch-type ECG sensor, composed of three Ag electrodes that are screen-printed on a flexible (but not soft) polymer (poly(ethylene terephthalate)) substrate and covered by commercially available gel sheets (total thickness: few mm) for conformal contact with skin [15]. This sensor was attached on a skin surface of a subject with the aid of a medial glue sheet and recorded ECG signals continuously while the subject did physical exercise. From the ECG signal, the heart rate (i.e., the intervals between R waves as indicated in the ECG-time plot of Figure 2B) was extracted, which can be used to monitor the evaluation of stress level during the exercise.

Many wearable ECG electrodes need to be wired to bulk or small portable instrument for data acquisition as shown in the photo of Figure 2B, restricting the mobility of the subject. Recent technological advances in wireless communication between electronic devices have realized wireless ECG sensing, which will greatly improve the applicability of wearable ECG systems. For instance, ECG electrodes can be formed on one side of a flexible substrate where an integrated circuit including Bluetooth module for wireless data transmission to a mobile device is mounted on the other side [16]. Other wireless technologies (e.g., for power transfer) are also required to realize stand-alone wearables, which is discussed in a recent review [17].

In the case of patch-type ECG devices, it is challenging to secure the robust electrode–skin contact in long term. The ECG signal in Figure 2B shows gradual attenuation, possibly due to the increased contact impedance resulting from the deterioration of contact integrity by sweat secretion, relative motion between electrode and skin, and the gel dehydration. Dried gel also causes irritation, which reduces long-term adherence of the subject to ECG patches.

Thinner and softer electrodes allow more intimate coupling with skin. Such electrodes will enlarge the contact area, consequently lowering the contact impedance and minimize the shear force exerted to the electrode–skin interface, suppressing some artificial signals caused by motion. Electrodermal electronics (also called e-tattoos), which are composed of thin (few to tens of micrometer thick), skin-soft electrodes, have recently emerged as promising candidates for wearable ECG electrodes. Schematics in Figure 2C(C-i) illustrate one of the typical methods to fabricate and transfer an e-tattoo [18]; an electrode is patterned on a water-soluble tattoo paper, a small amount of water is applied to the skin, the electrode paper is pressed on the skin for a while, and the tattoo paper is peeled off, leaving the electrode adhered to the skin via van der Waals force. Another type of transferring method utilizes the difference of thermal expansion coefficients between the transfer substrate and e-tattoo [19]. The transferred electrode can conform with the morphology of microscopically rough skin surface as shown in the photo in Figure 2C(C-ii), and maintain its stable skin contact under localized skin motion. These electrodes captured ECG signals accurately as the conventional ECG sensor composed of the Ag/AgCl gel electrode (the voltage–time plot in Figure 2C(C-i)).

For an electrode in e-tattoos, mostly Au has been adopted due to its high electrical conductivity, biocompatibility, and the ease of fabrication into thin layers. This inevitably increases the manufacturing cost of e-tattoos that are usually used in a disposable manner. In this sense, other conducting materials that are thin and soft, such as conductive polymer (a blend of poly(ethylenedioxythiophene):poly(styrenesulfonate) (PEDOT-PSS), waterborne polyurethane, and D-sorbitol) [20] and graphene [21], are being investigated as ECG electrodes.

To maximize the conformality of wearable ECG sensors to skin morphology, a paint-on-type sensor has been recently developed [22]. As illustrated in Figure 2D, conductive ink (the mixture of Ag flakes and a commercially available nontoxic poly(vinyl alcohol)-based glue) was directly sprayed onto the skin masked by a patterned adhesive, molded onto the skin with features (e.g., ridge), and left for few minutes to dry, which was expedited by the body heat. When the dried film that was intentionally delaminated from the skin, skin-like roughness of the film surface was observed, which confirmed the intimate film-skin contact. This painted electrode was connected to an ECG recording instrument via bare wires and successfully captured ECG traces as the conventional electrode (the ECG-time plots in Figure 2D). After using, the painted electrode was easily removed from the skin by using a tape.

The abovementioned ECG sensors show various approaches to improving the electrical, mechanical, and biological properties of the electrodes that are directly attached to skin. ECG sensors can be embodied in garments by using conductive textile that work as electrodes. Photos in Figure 2E show the thread electrodes that are coated with Ag [23] or conducting polymer (PEDOT-PSS) [24], sewn into fabrics, and connected to the signal acquisition device via metallic snap buttons. The conductive textile maintains its electrical conductivity against 50 times of washes. When worn properly (i.e., fitted tightly to human chest), this textile-based sensor records ECG signals which are comparable to those recorded by conventional gel electrodes.

## 3. Wearable Sensors for Monitoring Respiratory Activity

**Figure 3:**
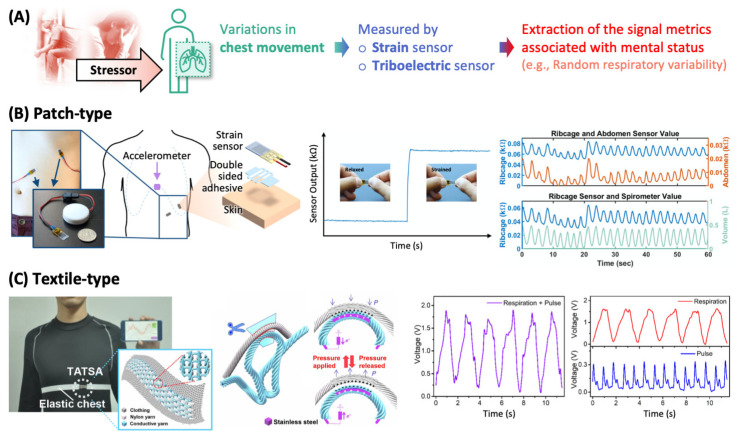
Wearable sensors for monitoring respiratory activities. (**A**) Mental stress-relevant respiratory activities can be monitored by measuring chest movement. (**B**) Piezoresistor-based strain sensor patch and (**C**) conducting textile-stitched sensor strap for monitoring chest movement during respiration.

Respiration is regulated by ANS to meet metabolic demands for maintaining physiological homeostasis and to respond to mental stressors [25,26]. It has been reported that the respiratory patterns, which are characterized by respiration rates, inspiration/expiration ratios, respiratory pauses, abdominal/thoracic respiration ratios, respiratory volume, irregularity, etc., are affected by various mental stressors (e.g., anger, anxiety, depression, fear, and sadness) and are individually specific. For instance, the respiration rate increases when an individual experiences a panic attack and the increment is dependent on the individual’s baseline level of anxiety [27]. By monitoring respiratory patterns, it is possible to objectively and quantitatively evaluate the mental state. There are several approaches to obtaining respiratory patterns and many of them utilize chest movements during respiration (Figure 3A); this changes the electrical properties (e.g., bioimpedance, not covered in this review) and physical properties (e.g., circumference) of chest region.

During inhalation and exhalation, the circumferences of the thorax and abdomen vary, and the strain sensors attached to such regions can detect such circumference variation as the sensors stretch and relax along with the underlying skin. Figure 3B shows the photo of piezoresistor-based strain sensors placed around thorax (ribcage) and abdomen. The piezoresistive component is thin metal film (5 nm of Pt and Au) adhered to an elastic substrate (silicone elastomer) that conforms to a movable object [28]. When mechanical strain is transferred through the underlying elastomer to the metal film, the film undergoes fracture, and its electrical resistance increases as shown in the sensor output–time plot in Figure 3B. When these strain sensors are attached perpendicular to the thorax and abdomen areas to minimize crosstalk, they generate electrical resistance signals that oscillate by the subject’s respiration (the upper co-plot of ribcage and abdomen vs. time in Figure 3B). Their waveforms are consistent with each other and coincident with that of the variation in lung volume measured by a spirometry, a conventional method for analyzing respiration (the lower co-plot of ribcage and volume vs. time in Figure 3B). This set of wearable strain sensors can be used to calculate the rate and volume of respiration (and other metrics of respiratory patterns) for continuous monitoring of mental health.

A piezoresistor-based strain sensor for monitoring respiration is built on elastomeric system and undergoes stretch–relaxation cycles to conform with repetitive chest movement. The viscoelastic properties of polymeric substrates inherently have hysteresis in cyclic strain, which limits the sensitivity for detecting chest movement. In the material standpoint, the optimal interfacial adhesion between the piezoresistor and the elastomeric substrate can provide more appropriate hysteresis behavior [29].

Chest movement can be detected via a triboelectric effect of smart garments composed of conductive textile. For instance, the conductive yarn, made of stainless-steel core fiber wrapped in poly(ethylene terephthalate) fiber, and typical nylon yarn are knitted together and stitched into an elastic chest strap where electronics for signal processing and transmission to a mobile phone are also attached, forming a wireless chest strain sensor (see photo in Figure 3C) [30]. When this strap is worn by a subject, and as the chest volume expands and contracts during the respiration, external pressure is applied and released to the interfacial surface between the conductive and nylon yarns. The contact–separation movement between the two yarns induces triboelectrification at the interface. And then when the two yarns are separated, charge is induced in the stainless-steel core fiber of the conductive yarn via electrostatic induction as illustrated in the schematics of Figure 3C. This electricity-generating process can be simply observed by measuring the output voltage generated in the conductive yarn. The output voltage signal, however, contains information on not only the respiration but also heart beating; the signals originating from two processes are superimposed (the left voltage–time plot in Figure 3C). As respiration contributes to the chest volume variation at larger amplitude and lower frequency than heart beating does, it is possible to extract the respiratory information from the output signal by using a low-pass filter; the heartbeat signal can be extracted by a band-pass filter (the right plots in Figure 3C).

## 4. Wearable Sensors for Monitoring Perspiratory Activity

**Figure 4:**
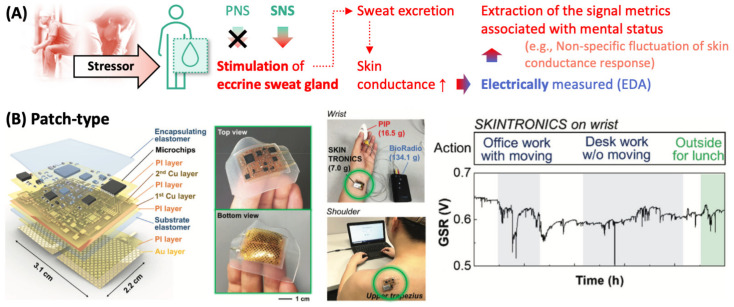
Wearable sensors for monitoring perspiratory activities. (**A**) Mental stress-relevant perspiratory activities can be monitored by EDA. (**B**) Electrodermal activity sensor fabricated on a silicone-based elastomer patch. PNS, parasympathetic nervous system; SNS, sympathetic nervous system; and EDA, electrodermal activity.

Sweat is a biofluid produced by eccrine and apocrine sweat glands. Eccrine glands are found almost all over the body, with high densities in palms, soles, and forehead, functioning as major sweat glands; apocrine glands are found locally (e.g., armpit). Importantly, eccrine glands are stimulated exclusively by the sympathetic branch, which is activated under stressful situations, of ANS (Figure 4A). The level of sweat secretion, therefore, is an ideal biomarker of SNS activation (and, consequently, of the reaction to stressors).

As sweat contains ions (e.g., Na^+^, Cl^−^, K^+^), small molecules (e.g., urea, pyruvate/lactate), and even macromolecular substances such as proteins and is electrically conductive, its secretion can be monitored by measuring the electrical conductance of skin, so called electrodermal activity (EDA) or galvanic skin response (GSR); skin has electrical resistance of ~100 kΩ and as sweat is secreted the skin resistance decreases [31]. EDA is characterized by a slowly changing signal (i.e., skin conductance level) and fast-changing signal (i.e., skin conductance response). Interestingly, it has been shown that EDA can be used to indicate the course of body reactions to mental stressors (e.g., anxiety, fear, and high cognitive load) [32]; for instance, the non-specific fluctuation of skin conductance response indicates the emotional component of the body reaction [33].

An EDA signal can be obtained from the two electrodes attached to skin surface; the voltage between them is measured (without the application of external current), or a small current is passed through them and then the output voltage is measured. As there is a high concentration of eccrine sweat glands in fingers, a ring-type sensor was developed for continuous EDA monitoring [34]. A prototype of the finger-worn EDA sensor has inner radius of 15~18 mm, outer radius of 16.5~19.5 mm, and constant thickness of 1.5 mm, and contains two metal contacts composed of flexible Ag material and other electronic components (e.g., battery) for wireless sensor operation. To test the capability of a ring-type EDA sensor for monitoring mental status, student volunteers wore the rings and then took exams while the ring sensors continuously collected EDA signals. A drastic change in EDA signal was observed, indicating the elevation of stress level during exams. Interestingly, 86% of the subjects preferred this wireless ring-type sensor to a watch-type sensor in terms of comfortableness and wearability.

Many wearable EDA sensors are placed on the palms, fingers, or soles to maximize the sensitivity in monitoring the perspiratory activity. This, however, poses some limit in their applicability because such sensor attachment is obtrusive, inevitably hinders daily activities, and is vulnerable to detachment. In this sense, there has been a development of EDA sensors that target nonpalmar and nonplantar regions [35], and an example of such wearable EDA sensor is shown in Figure 4B [36]. A pair of metal mesh electrodes are patterned on a silicone-based elastomeric substrate, connected to microchips (composed of EDA measurement circuit, microcontroller, and analogue-to-digital converter) and the microchip is encapsulated with another elastomer film, leaving the electrodes exposed at the bottom of the sensor. This flexible and stretchable EDA sensor can be mounted on wrist or shoulder (photos in Figure 4B) by simply pressing the sensor without the aid of adhesive gels or tapes. It provides conformal and comfortable interface between electrodes and skin without irritation or redness for over 3 h. It also provides robust electrical signal (i.e., negligible change of output voltage) even when it undergoes up to 20% tensile strain; this ensures the robustness of sensor operation against the natural deformation of underlying skin. (Note: Skin can tolerate tensile strain up to 20% [37].) The feasibility of this EDA sensor for continuous stress monitoring in daily activities was tested by mounting the sensor on a subject’s wrist and recording the output voltage while the subject conducted office work or walked outside (the GSR-time plot in Figure 4B). The voltage peak indicates an arousal status of SNS and the number of peaks per minute can be used to quantify the subject’s stress level.

## 5. Wearable Sensors for Detecting Molecular Biomarkers

**Figure 5:**
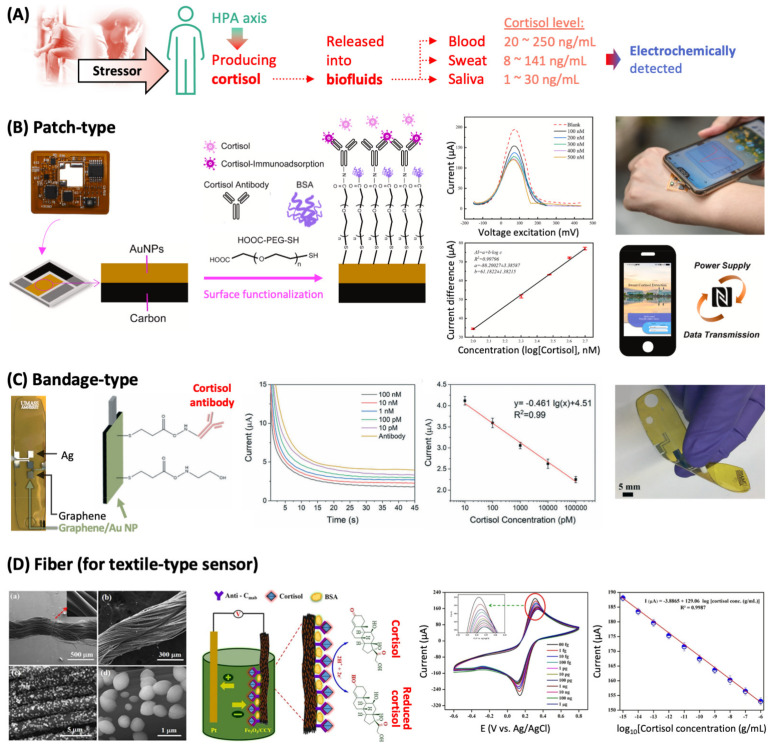
Wearable electrochemical immunosensors for detecting cortisol (stress hormone). (**A**) Mental stress-relevant molecular secretions into biofluids can be monitored by electrochemical methods. (**B**) Patch-type sensor fabricated on a flexible poly(ethylene glycol terephthalate) substrate. The sensor chip contains near field communication module for wireless energy supply and wireless data transmission. (**C**) Bandage-type sensor with inkjet-printed electrode on a polyimide substrate. (**D**) Cortisol-detecting conductive fiber. HPA, hypothalamic–pituitary–adrenal; BSA, bovine serum albumin; and PEG, poly(ethylene glycol).

Physiological changes are mediated by specific biomolecule such as ions (e.g., Na^+^ and K^+^), hormones (e.g., cortisol and adrenaline), and neurotransmitters (e.g., neuropeptide Y and gamma-aminobutyric acid), and they have been used as molecular biomarkers to examine health status. Cortisol, in particular, has been employed as a stress-related biomarker. It is a hormone that is released from the adrenal cortex and reaches multiple body fluids, which is triggered by the stimulation of the HPA axis, the endocrine core of stress system (Figure 5A). Cortisol secretion temporarily increases the availability of energy by affecting muscle strength, memory function, immunity, and the sensitivity to pain to cope with stressors [38]; this increased cortisol secretion is terminated via a feedback-loop to maintain homeostasis of the body functions. The abnormalities of HPA axis result in hyper- and hypocortisolisms, and it has been revealed that such dysfunctions are connected with mental illnesses such as depression and post-traumatic stress disorder, respectively [39]. Therefore, by monitoring the level of secreted cortisol, it is possible to obtain some information on metal state.

As the molecular biomarkers are generally present at “low concentrations” in the “intrinsically crowded biofluids” (e.g., sweat, saliva, tears, urine, and blood), it is essential for the sensors to have appropriate detection sensitivity and selectivity. There are numerous strategies to improve the detection sensitivity, which will not be covered in this review. Detection selectivity can be acquired by adopting target recognition units, also called bio-receptors, which is discussed further below. Molecular biosensors can be categorized in terms of the measurement modality: optical (e.g., absorption, photoluminescence, and Raman scattering), mechanical, electrical (e.g., using field-effect transistors), and electrochemical modes. The electrochemical mode, particularly, offers various advantages in realizing biomolecular detection in a wearable form, mainly because the instrument for electrochemical measurement is simply constituted and easily miniaturized. In this review, we introduce some of the recently reported electrochemical sensors that target cortisol in sweat.

Table 1 summarizes the important properties of some electrochemical cortisol sensors, which are categorized by their recognition units: antibody, aptamer, and molecularly imprinted polymer (MIP). Antibody is the most widely used recognition unit due to the high affinity and specificity of its binding with the target substance, and any sensors that utilize antibody are called immunosensors. Aptamer is a short, single- or double-stranded nucleic acid, either DNA or RNA, that has a high affinity to a certain substance (ranging from ions, small molecules, nucleic acids, proteins, virus, and bacteria to human cells); the aptamer-based biosensors are called aptasensors. The specific sequence of aptamer bases enables the aptamer strand to form a unique three-dimensional morphology that fits to the target substance via various intra- and intermolecular interactions (e.g., hydrogen bonding, dipole–dipole interaction, or π–π interaction). Due to its higher chemical stability, versatility in interaction with different target substances, and easier production (i.e., chemical synthesis) compared with antibodies, aptamer has been established as one of the preferred recognition units. MIP is a synthetic polymer that is polymerized in the presence of a target substance, which functions as a template, followed by the removal of the target substance, leaving behind the molecularly imprinted cavities that fit to the target [40]. There are several antibodies, aptamers, and MIPs that specifically capture cortisol, and they are employed to functionalize electrodes to sense cortisol.

Electrochemical cortisol immunosensors with different form factors are shown in Figure 5. Their structures are different, but all of them have a three-electrode system consisting of working, reference, and counter electrodes for reliable measurement.

The most common form adopted for wearable electrochemical sensors is based on a patch. Figure 5B shows the photo and fabrication schematics of a patch-type sensor that has a flexible substrate (poly(ethylene glycol terephthalate)) where Ag/AgCl and carbon were printed to act as reference and counter electrodes, respectively, and Au nanoparticles (NPs) were deposited to form a working electrode [41]. Au NPs were functionalized with cortisol antibody via a linker molecule (poly(ethylene glycol) with carboxyl and thiol groups at each end, HOOC-PEG-SH). The region uncovered with cortisol antibody was inactivated by bovine serum albumin (BSA, a common inactivating agent). A solution containing freely diffusing redox probe (e.g., a water-soluble derivative of ferrocene) was dropped on the as-fabricated sensor patch, and the strong electrochemical signal of the probe was obtained. When a sample solution containing cortisol was spiked with the redox probe and dropped on the sensor, cortisol was captured by the antibody, placed in close proximity to the electrode surface, and resultantly hindered the diffusion of the probe toward the electrode surface (the current–voltage excitation plot in Figure 5B). As a result, the probe signal decreased linearly with the increase in cortisol concentration (the current difference–cortisol concentration plot in Figure 5B). The limit of detection (LOD) of 7.47 nM was achieved, which is acceptable to detect cortisol in sweat (c.f., physiological cortisol level in sweat: 1.4 nM–0.4 μM). To demonstrate the practical applicability of the sensor patch, the patch was attached to the arm of the subject who had excreted sweat, infiltrated by sweat, the redox probe was added to the patch, and the electrochemical signal was collected followed by the conversion of the signal to the cortisol concentration. The antibody-containing detection component of the patch can be disassembled after use, and a new component can be put in the patch for the next cortisol detection on demand.

Cortisol detection electrodes can be printed on a flexible bandage as shown in Figure 5C [47]. Ag and graphene inks were printed on a polymeric substrate (polyimide) to form reference and counter electrodes, respectively. On another printed graphene, Au NPs were electrodeposited to form a graphene/Au NP working electrode, followed by the covalent attachment of cortisol antibody to the Au NPs. When this bandage sensor is exposed to sweat that contains cortisol, cortisol is bound to the antibody and works as diffusion blocker. This linearly suppresses the electrochemical signal of a redox probe Fe(CN)_6_^3−/4−^ with increasing cortisol concentration, which can be used to quantify the cortisol concentration (plots in Figure 5C). This sensor bandage maintained its structural integrity during large mechanical deformation as shown in the photo in Figure 5C.

Textile-type cortisol sensor can be made from the fiber functionalized with cortisol antibody (Figure 5D) [45]. To functionalize conductive carbon yarn (i.e., working electrode), it was first coated with Fe_2_O_3_ NPs (see the scanning electron microscope images in Figure 5D) that facilitates the immobilization of cortisol antibodies via electrostatic interaction. The as-fabricated antibody/Fe_2_O_3_/conductive carbon yarn sensor (before being exposed to a cortisol-containing solution) showed a pair of redox peaks originating from Fe_2_O_3_. When cortisol was present in the analyte solution, the sensor’s antibody captured cortisol and the charge transfer resistance of the electrode coat increased as a result. This decreased the redox peak intensities with the increment of cortisol concentration (plots in Figure 5D). This functionalized conductive fiber has potential as the sensing component of smart garments that monitor the levels of stress hormones in sweat.

Wearable electrochemical sensors for detecting molecular biomarkers suffer from several limitations in common. Fundamentally, in electrochemical measurements, the diffusion of an electrochemically active substance (either target or redox probe) from the bulk solution (e.g., sweat) to the solution–working electrode interface and the electron transfer between the substance and the working electrode are the key processes. The material properties of a working electrode and the electrode’s functional coat significantly determine the sensor performances. One of the critical issues is the electrode fouling caused by non-specific adsorption of various biomolecules existing in biofluids, which blocks electron transfer at the electrode surface and deteriorates sensor sensitivity. This can be partially alleviated by using a semi-permeable electrode coat (e.g., cationic membrane such as Nafion) that can filter some of the biofluid substances (e.g., anionic substances, bulky substances), but not all [52].

Another significant concern is related to the functional coat of the sensing electrode. Many recognition units require redox mediators to transduce the target recognitions to electrochemical signals. This needs the supply of redox probe to the sensor, which increases the complexity of sensor operations (e.g., the sensor shown in Figure 5B requires a drop of the redox probe solution on the working electrode), hindering the true continuous measurement. This inconvenience can be partially resolved with the aid of sophisticated microfluidic technologies that can actively and continuously carry functional solutions (e.g., the sensor shown in Figure 5C contains electrowetting valves that electrically control the injection of the redox probe solution to the working electrode area). This method, however, also requires the supplies of the redox probe solution for continuous operation of the sensor if the sensor does not have an additional storage for the solution. Some recognition units are capable of generating electrochemical signals in response to their target without the addition of redox probe solution. For instance, a redox-probe-labeled aptamer changes its conformation upon target recognition, which pushes the redox probe toward the electrode surface resulting in the increase in electrochemical signal [53]. The aptamer without a redox label can also change the electrochemical signal, here electrochemical impedance, by the aptamer–target binding that increases the charge transfer resistance at the electrode–solution interface [54]. This sort of recognition unit has shown the opportunity for continuous, real-time molecular sensing for a prolonged time.

## 6. Opportunities and Challenges

Wearable sensors have a great potential as objective and continuous monitoring systems for mental health which so far has been examined by subjective and time-consuming methods (e.g., interview). Various body reactions to stressors can be detected by the sensors applied to human body for observing the variations in cardiac activity, respiration pattern, sweat secretion, and/or the secretion of stress hormones. The properties of interfaces between the sensor and the skin have a great effect on the quality of the measured biosignals and also on the user’s adherence to the sensor worn. Various approaches for forming the interface of high quality have been developed and, correspondingly, new sensor materials with improved properties have been developed.

Although there have been innovative advancements in various aspects, many wearable sensors still require the connection to bulky instruments for signal acquisition/processing, data storage/transmission, and power supply; this weakens the sensor’s wearability and thus user’s adherence to sensors. To solve this problem, wireless communication component (e.g., Bluetooth, Wi-Fi, near field communication (NFC)) and wireless power supply component (e.g., NFC) can be integrated to the sensor device (e.g., the patch-type cortisol sensor shown in Figure 5B contains NFC module).

In general, the sensor performance can be further improved by adopting multimodal measurements; this is especially valid in examining the state of high complexity, such as mental status. When multiple orthogonal biosignals (e.g., “ECG and EEG” or “EEG and PPG”) are used, the analytic capacity for monitoring mental status (e.g., anxiety or mental fatigue, respectively) can be improved [55,56]. In addition, a multimodal sensor system can be used to catch motion artifacts. For instance, the strain sensor for monitoring the respiratory activity (shown in Figure 3B) is sensitive to not only the chest movement, but also the torsion of torso; the accelerometer attached near the strain sensor can detect such motion artifacts. Information-rich data from the wearable multimodal sensor and the data analysis technologies strengthened by artificial intelligence will greatly improve the monitoring of mental status [8].

## Figures and Tables

**Table 1:** Wearable electrochemical sensors detecting cortisol.

Recognition Units	Electrode Materials	Electrochemical Techniques	Detection Limits	Form Factors	Ref.
Antibody	Au NP ^b^-modified carbon SPE ^c^	DPV ^g^	7.47 nM	Patch	[41]
	3D nanostructured Au	EIS ^h^	1 pg/mL	Patch	[42]
	LIG ^d^	Amp ^i^	0.08 ng/mL	Patch	[43]
	Mxene-loaded LIG	EIS	3.88 pM	Patch	[44]
	Fe_2_O_3_-modified carbon yarn	CV ^j^	0.005 fg/mL	Fiber	[45]
	TiO_2_-modified carbon yarn	DPV	6 fg/mL	Fiber	[46]
	Au NP-modified graphene IPE ^e^	Amp	10 pM	Bandage	[47]
Aptamer	CNT ^f^ composite	CV	1.8 ng/mL	Patch	[48]
	ZnO	EIS	2 ng/mL	Patch	[49]
MIP ^a^	C SPE	EIS	-	Patch	[50]
	CNT composite	EIS	2.0 ng/mL	Patch	[51]

^a^ MIP, molecularly imprinted polymer; ^b^ NP, nanoparticle; ^c^ SPE, screen-printed electrode; ^d^ LIG, laser-induced graphene; ^e^ IPE, inkjet-printed electrode; ^f^ CNT, carbon nanotube; ^g^ DPV, differential pulse voltammetry; ^h^ EIS, electrochemical impedance spectroscopy; ^i^ Amp, amperometry; and ^j^ CV, cyclic voltammetry.
